# Supplementary material for: Resolving the full spectrum of human genome variation using Linked-Reads
Source: Genome Res. 2019 Apr;29(4):635–45. doi: 10.1101/gr.234443.118 (PMC6442396; doi:10.1101/gr.234443.118)
Supplement: Supplemental Material [file supp_gr.234443.118_Supplemental_longranger-2.2.zip › longranger-2.2.tar.bz2!/lr2.2-public/lib/stan/cmdstan/stan/doc/api/html/check__positive_8hpp_source.html]

Stan: src/stan/math/error\_handling/check\_positive.hpp Source File


|  |  |
| --- | --- |
|  | Stan  2.5.0  probability, sampling & optimization |


- Main Page
- Related Pages
- Namespaces
- Classes
- Files

- File List
- File Members

All Classes Namespaces Files Functions Variables Typedefs Enumerations Enumerator Friends Macros Pages

- src
- stan
- math
- error\_handling

check\_positive.hpp

Go to the documentation of this file.

1 #ifndef STAN\_\_MATH\_\_ERROR\_HANDLING\_CHECK\_POSITIVE\_HPP

2 #define STAN\_\_MATH\_\_ERROR\_HANDLING\_CHECK\_POSITIVE\_HPP

3

4 #include <boost/type\_traits/is\_unsigned.hpp>

5 #include <stan/math/error\_handling/dom\_err.hpp>

6 #include <stan/math/error\_handling/dom\_err\_vec.hpp>

7 #include <stan/math/meta/value\_type.hpp>

8 #include <stan/meta/traits.hpp>

9

10 namespace stan {

11

12  namespace math {

13

14  namespace {

15

16  template <typename T\_y, typename T\_result, bool is\_vec>

17  struct positive {

18  static bool check(const char\* function,

19  const T\_y& y,

20  const char\* name,

21  T\_result\* result) {

22  // have to use not is\_unsigned. is\_signed will be false

23  // floating point types that have no unsigned versions.

24  if (!boost::is\_unsigned<T\_y>::value && !(y > 0))

25  return dom\_err(function,y,name,

26  " is %1%, but must be > 0!","",

27  result);

28  return true;

29  }

30  };

31

32  template <typename T\_y, typename T\_result>

33  struct positive<T\_y, T\_result, true> {

34  static bool check(const char\* function,

35  const T\_y& y,

36  const char\* name,

37  T\_result\* result) {

38  using stan::length;

39  for (size\_t n = 0; n < length(y); n++) {

40  if (!boost::is\_unsigned<typename value\_type<T\_y>::type>::value

41  && !(stan::get(y,n) > 0))

42  return dom\_err\_vec(n,function,y,name,

43  " is %1%, but must be > 0!","",

44  result);

45  }

46  return true;

47  }

48  };

49

50  }

51

52  // throws if any element in y is nan

53  template <typename T\_y, typename T\_result>

54  inline bool check\_positive(const char\* function,

55  const T\_y& y,

56  const char\* name,

57  T\_result\* result) {

58  return positive<T\_y,T\_result,is\_vector\_like<T\_y>::value>

59  ::check(function, y, name, result);

60  }

61

62  }

63 }

64 #endif

dom\_err.hpp

stan::math::dom\_err

bool dom\_err(const char \*function, const T &y, const char \*name, const char \*error\_msg, const T\_msg error\_msg2, T\_result \*result)

**Definition:** dom\_err.hpp:36

stan::length

size\_t length(const T &)

**Definition:** traits.hpp:159

stan::math::dom\_err\_vec

bool dom\_err\_vec(const size\_t i, const char \*function, const T &y, const char \*name, const char \*error\_msg, const T\_msg error\_msg2, T\_result \*result)

**Definition:** dom\_err\_vec.hpp:33

traits.hpp

value\_type.hpp

stan::math::check\_positive

bool check\_positive(const char \*function, const T\_y &y, const char \*name, T\_result \*result)

**Definition:** check\_positive.hpp:54

dom\_err\_vec.hpp

stan::get

T get(const T &x, size\_t n)

**Definition:** traits.hpp:143


---

[ Stan Home Page ]

*© 2011–2014,
Stan Development Team.*
